# Acquisition of drug resistance in endothelial cells by tumor-derived extracellular vesicles and cancer progression

**DOI:** 10.20517/cdr.2023.121

**Published:** 2024-01-05

**Authors:** Masahiro Morimoto, Nako Maishi, Kyoko Hida

**Affiliations:** ^1^Department of Vascular Biology and Molecular Pathology, Hokkaido University Faculty of Dental Medicine, Sapporo 060-8586, Japan.; ^2^Department of Oral Diagnosis and Medicine, Hokkaido University Faculty of Dental Medicine, Sapporo 060-8586, Japan.

**Keywords:** Tumor endothelial cell, drug resistance, extracellular vesicles, miRNA

## Abstract

Angiogenesis by endothelial cells (ECs) is essential for tumor growth. Angiogenesis inhibitors are used in combination with anticancer drugs in many tumor types, but tumors eventually become resistant. Previously, the underlying mechanism for developing drug resistance was considered to be a change in the characteristics of tumor cells whereas ECs were thought to be genetically stable and do not contribute to drug resistance. However, tumor endothelial cells (TECs) have been shown to differ from normal endothelial cells (NECs) in that they exhibit chromosomal abnormalities, angiogenic potential, and drug resistance. Extracellular vesicles (EVs) secreted by tumor cells have recently attracted attention as a factor involved in the acquisition of such abnormalities. Various cells communicate with each other through EVs, and it has been reported that tumor-derived EVs act on other tumor cells or stromal cells to develop drug resistance. Drug-resistant tumor cells confer drug resistance to recipient cells by transporting mRNAs encoding ATP-binding cassette subfamily B member 1 (ABCB1) and ATP-binding cassette subfamily C member 1 (ABCC1) as well as miRNAs involved in signaling such as Akt, drug efflux transporters, and P-glycoprotein modulators via EVs. However, there are limited reports on the acquisition of drug resistance in ECs by tumor-derived EVs. Since drug resistance of ECs may induce tumor metastasis and support tumor cell proliferation, the mechanism underlying the development of resistance should be elucidated to find therapeutic application. This review provides insight into the acquisition of drug resistance in ECs via tumor EVs in the tumor microenvironment.

## INTRODUCTION

### Tumor angiogenesis and antiangiogenic therapy

Angiogenesis refers to the sprouting and elongation of new vascular branches from existing vessels, and vasculogenesis refers to the process by which vascular endothelial cells (ECs) differentiate from vascular progenitor cells to form a lumen^[1]^. Angiogenesis is induced in ischemic tissues, such as tumors and wound healing tissue, when cells release angiogenic factors such as vascular endothelial growth factors (VEGF) under the influence of hypoxia.

Angiogenesis by ECs is essential because tumor growth requires the supply of nutrients and oxygen from blood vessels^[2]^. Tumor blood vessels also provide a route for tumor cells to metastasize to distant organs^[3,4]^. Therefore, tumor blood vessels have been recognized as an important target for cancer treatment since Dr. Folkman proposed the idea that tumor growth depends on angiogenesis in 1971^[5]^. Antiangiogenic therapy blocks angiogenesis, a common process in tumor growth. The first angiogenesis inhibitor was bevacizumab, a monoclonal antibody against human VEGF. In addition, sorafenib, sunitinib, and pazopanib, which inhibit the VEGF receptor (VEGFR) tyrosine kinase, have been developed for cancer treatment^[6]^. Angiogenesis inhibitors also normalize immature, leaky, and dysfunctional tumor blood vessels and improve the delivery of drugs and immune cells to tumor tissues^[7,8]^. Thus, angiogenesis inhibitors displayed an additive effect when combined with anticancer drugs such as 5-FU, cisplatin, and gemcitabine in many tumor types^[9]^. However, existing angiogenic inhibitors, such as bevacizumab, block VEGF signaling, which is also essential for normal blood vessels. These drugs may cause side effects such as hypertension and bleeding^[10]^.

### The abnormalities and drug resistance of tumor endothelial cells

Tumor blood vessels are histo-pathologically different from normal vessels^[11]^. Vascular permeability is increased due to sparse adhesion between ECs and between mural cells and ECs^[12]^. The vascular basement membrane is also abnormal, showing intermittent vessel bending, which may cause staggered blood flow^[8]^. In other words, tumor vessels are heterogeneous and disorganized in contrast to normal blood vessels that have an ordered, hierarchical structure^[13]^. Therefore, despite the abundance of blood vessels, blood flow is low and cancer tissue is hypoxic^[14]^.

Angiogenesis inhibitors used in combination with anticancer drugs are effective in the early stages, but tumors eventually become resistant, which limits the long-term therapeutic use of these drugs. One of the advantages of targeting ECs rather than tumor cells is that, unlike tumor cells, which are genetically unstable, ECs were thought to be genetically stable and do not acquire drug resistance until recently^[15]^. The mechanism underlying the development of resistance has been considered to be a change in the characteristics of tumor cells. These include the acquisition of anticancer drug resistance by tumor cells^[16]^ and the increased production of angiogenic factors other than VEGF [e.g., fibroblast growth factor (FGF)] as a compensatory response to VEGF inhibition^[17]^. However, in the tumor microenvironment, tumor endothelial cells (TECs) are exposed to hypoxia, nutrient deprivation, and cytokines secreted by tumor cells and tumor stromal cells. In other words, TECs exist in a very different environment compared to normal endothelial cells (NECs). These are all known as mechanisms by which cancer cells acquire drug resistance. In fact, TECs exhibit a variety of abnormalities^[18]^. We found that TECs have various abnormalities compared to NECs, including chromosomal abnormalities^[19,20]^, increased angiogenic potential^[21]^, and elevated expression of genes such as cyclooxygenase-2^[22]^, VEGF^[23]^, CXCR7^[24,25]^, biglycan^[26-28]^, lysyl oxidase^[29]^, lectin-like ox-LDL receptor 1^[30]^, and PTGIR^[31]^. In addition, we have reported that tumor cell-secreted VEGF and other factors upregulate the expression of MDR1/ATP-binding cassette subfamily B member 1 (ABCB1), a stem cell marker, in TECs. The ABC transporter P-glycoprotein (P-gp), a transcript of MDR1/ABCB1, promotes drug efflux^[32,33]^. We also found that inflammatory changes in tumor tissues during anticancer therapy, such as increased IL-8 production, induce MDR1/ABCB1 expression in TECs, resulting in resistance to paclitaxel, which is mediated by ABCB1^[34]^. Naito *et al.* reported the existence of stem cell-like TECs that express high levels of P-gp, a stem cell marker, and are resistant to tyrosine kinase inhibitors that primarily target VEGFR^[35]^. We found that the stem cell markers aldehyde dehydrogenase (ALDH), Sca-1, CD90, and MDR1 were highly expressed in TECs, indicating drug resistance^[36]^. Furthermore, TEC in renal carcinoma was shown to be resistant to vincristine^[37]^ while TEC in hepatocellular carcinoma is resistant to 5-FU^[38]^. These findings suggest that TECs that have acquired stem cell-like properties in the tumor microenvironment are likely to survive after chemotherapy and maintain tumor cell proliferation. Therefore, understanding the properties and the mechanisms of characteristic changes of TECs is very important to study the effectiveness of drug treatment. It has been reported that drug resistance, chromosomal aberrations, and stemness of TECs differ depending on tumor malignancy^[39]^. These results suggest that factors derived from the tumor microenvironment may induce abnormalities in ECs. Paracrine effects of tumor microenvironment factors, such as tumor cell-derived cytokines, on ECs have been reported^[40]^. It was also suggested that hypoxia may cause abnormalities in ECs^[41]^. Furthermore, tumor cell-derived extracellular vesicles (EVs) may be involved in the development of drug resistance in ECs.

## EFFECTS OF TUMOR EVS ON STROMAL CELLS IN THE TUMOR MICROENVIRONMENT

### EVs as a tool for intercellular communication

EVs secreted by tumor cells have attracted attention as tumor microenvironment factors. EVs are vesicles with lipid bilayer membranes that are 50-1,000 nm in diameter and secreted by various cells^[42]^. They have been called exosomes, ectosomes, microvesicles, shedding vesicles, *etc.*, depending on their size and origin^[42]^. Exosomes are endosomal membrane-derived vesicles formed by endocytosis processes at 30-120 nm. The main components are lipids, proteins, and nucleic acids (miRNA, mRNA, DNA)^[43,44]^. In general, many exosomes contain the multivesicular body formation and transport-related proteins (ALIX, TSG101), tetraspanins (CD9, CD63, CD81), and heat shock proteins (HSP70, HSP90)^[45]^. These are recognized as specific markers for exosomes and are used for characterization. Microvesicles are 100-1,000 nm vesicles that, unlike exosomes, bud and are secreted directly from the cell membrane^[44]^. Although they have different biosynthetic mechanisms, they share many components and sizes with exosomes, and it is difficult to completely separate them. EV isolation methods include ultracentrifugation, ultrafiltration, and immunoaffinity^[46]^, while ultracentrifugation is the “gold standard” for EV isolation^[46]^. Each method differs in the accuracy of isolation, required time, cost, and scale, and it is necessary to select the most appropriate method according to the nature of the research. Currently, the International Society for Extracellular Vesicles (ISEV) recommends the use of EV as a generic term for vesicles secreted by cells. The Minimal Information for Studies of Extracellular Vesicles 2018 (MISEV2018)^[47]^ describes EV characterization as follows: (a) Quantification: since EVs themselves are difficult to quantify, both the source of EVs and the preparation of EVs should be described quantitatively; (b) General characterization: (i) Analyze at least three positive protein markers of EVs, including at least one transmembrane or lipid-bound protein - cytosolic protein; and (ii) Analysis of at least one negative protein marker expression; and (c) Characterization of single vesicles: Use two different but complementary techniques: (i) Electron microscopy or atomic force microscopy; and (ii) Single particle analyzers.

EVs differ from factors involved in intercellular communication, such as cytokines and extracellular matrix, in that they are complexes of different substances^[43]^. Information from the source cell is packaged into EVs and taken up by the recipient cell to exert various effects^[48]^. EVs secreted by tumor cells are known to act on the tumor itself and the tumor microenvironment to influence its growth and metastasis^[49]^. The contents of EVs exhibit characteristics of the cells from which they are derived. Therefore, EVs circulating in body fluids are used as liquid biopsies and are recognized as biomarkers for early detection, diagnosis, treatment, and response to treatment in cancer patients.

### Effects of tumor EVs on stromal cells

Stromal cells play an important role in the tumor microenvironment. The stromal cells are composed of vascular and lymphatic ECs, macrophages, dendritic cells, lymphocytes, neurocytes, fibroblasts, and adipocytes. It has been reported that tumor cells act on these stromal cells by secreting EVs to create an environment conducive to their own growth and metastasis. For example, miR-146a-5p in EVs secreted by osteosarcoma cells is taken up by macrophages (preosteoclasts) and inhibits differentiation into osteoclasts, promoting tumor invasion and metastasis^[50]^. miR-1247-3p in EVs secreted by highly metastatic hepatocellular carcinoma cells directly targets B4GALT3 in fibroblasts and activates β1-integrin-NF-κB signaling. As a result, inflammatory cytokines such as IL-6 and IL-8 are secreted, promoting lung metastasis^[51]^. Colorectal cancer-derived integrin beta-like 1 (ITGBL1)-rich EVs stimulate the NF-κB signaling pathway via tumor necrosis factor (TNF) alpha-induced protein 3 (TNFAIP3) to activate fibroblasts at metastatic sites and promote cancer metastasis^[52]^. EVs released from glioma cells, including Wilms tumor-1 (WT1), are taken up by microglia and downregulate the expression of thrombospondin-1, a negative regulator of angiogenesis. As a result, angiogenesis is promoted by microglia^[53]^.

### Promotion of angiogenesis by tumor EVs

**Figure 1:**
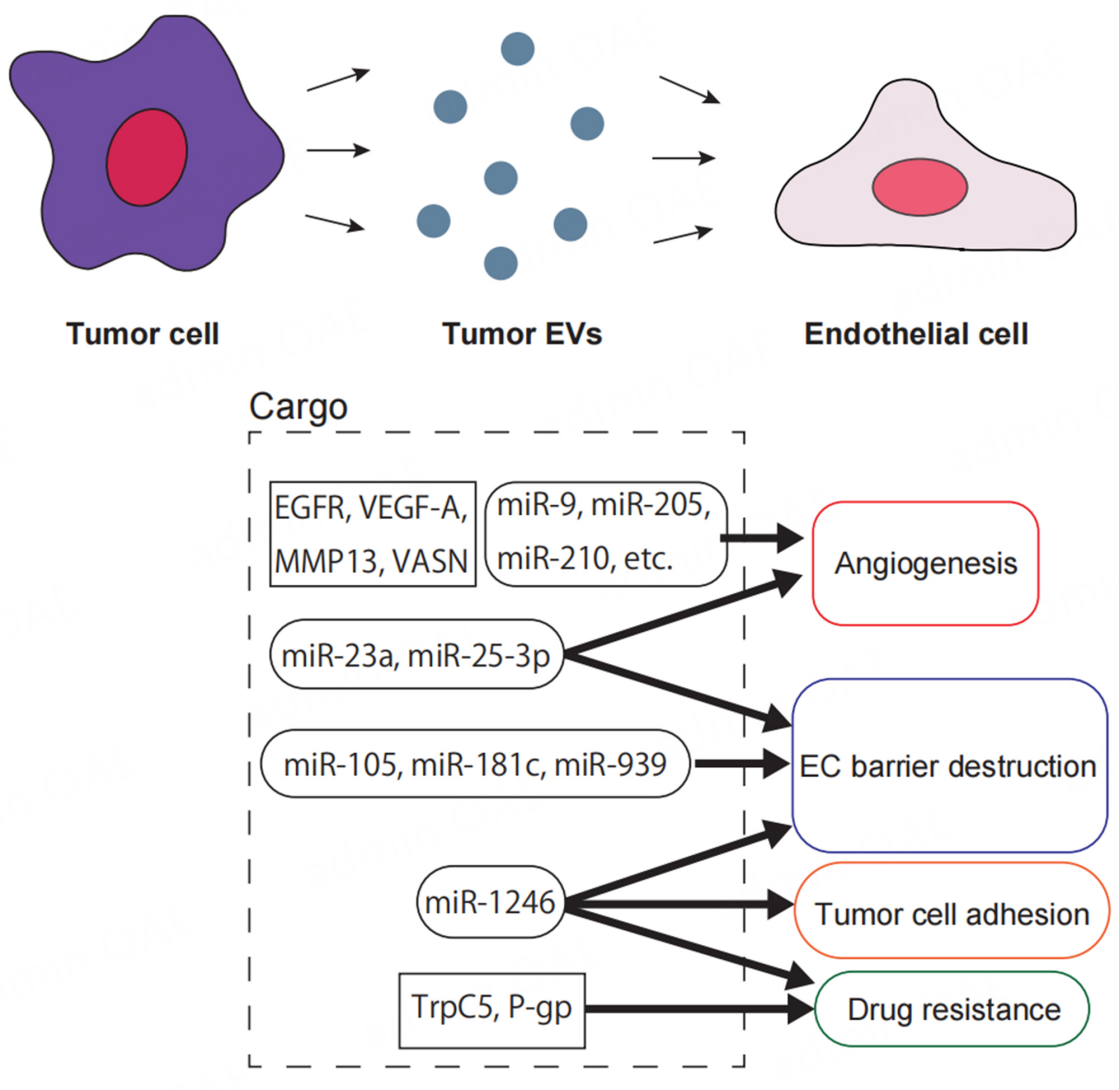
Tumor-derived EVs exert various effects on ECs. Tumor-derived EV cargo molecules such as EGFR, VEGF-A, MMP13, VASN, miR-9, miR-205, and miR-210 are transported to ECs to induce the proangiogenic phenotype. miR-105, miR-181c, and miR-939 attenuate endothelial adhesion. miR-23a and miR-25-3p induce angiogenesis and disrupt the EC barrier, while TrpC5 and P-gp confer drug resistance to ECs. In addition, miR-1246 promotes tumor cell adhesion to ECs, disrupts the EC barrier, and confers drug resistance. EVs: Extracellular vesicles; ECs: endothelial cells; EGFR: epidermal growth factor receptor; VEGF-A: vascular endothelial growth factor A; MMP13: matrix metalloproteinase 13; VASN: vasorin.

Tumor-derived EVs also act on ECs [Figure 1], and many reports have shown that they enhance angiogenic potential in particular. For example, EVs derived from human squamous cell carcinoma cells transport epidermal growth factor receptor (EGFR) to ECs and enhance their angiogenic potential^[54]^. In addition, vascular endothelial growth factor A (VEGF-A)^[55]^, matrix metalloproteinase 13^[56]^, and vasorin^[57]^ in tumor-derived EVs are transported to ECs and enhance their angiogenic potential.

miRNAs are also important as EV cargoes that induce angiogenesis. miR-210 in EVs derived from breast cancer^[58]^ or hypoxic leukemia cells^[59]^, and miR-9 in glioma EVs^[60]^ are transported to ECs and induce angiogenesis. In addition, we reported that tumor cell-derived EVs were incorporated into EC and enhanced their angiogenic potential via Akt activation^[61]^.

### Pre-metastatic niche formation by tumor EV

Furthermore, since ECs act as gatekeepers for tumor metastasis, altering their characteristics may promote metastasis. In particular, it has been reported that targeting adhesion molecules between ECs, such as VE-cadherin, ZO-1, and Claudin-5, to weaken their adhesion promotes tumor metastasis. Most of them are based on the mechanism of the mRNA repression by miRNAs in EVs. Zhou *et al.* reported that miR-105 in EVs derived from metastatic breast cancer cells suppressed the expression of ZO-1, which forms tight junctions between ECs, and disrupted the endothelial barrier to promote metastasis^[62]^. miR-25-3p in colon cancer cell-derived EVs induced angiogenesis and vascular permeability by targeting KLF2 and KLF4 of ECs and regulating the expression of VEGFR2, ZO-1, occludin and claudin-5, thus promoting liver and lung metastasis^[63]^. Tominaga *et al.* showed that miR-181c in EVs secreted by highly metastatic breast cancer cells downregulates expression of PDPK1 and inhibits actin polymerization in ECs, thereby disrupting the blood-brain barrier and promoting brain metastasis of breast cancer^[64]^. miR-939 in breast cancer cells downregulates VE-cadherin and destroys EC barrier^[65]^. Hsu *et al.* reported that miR-23a in EVs secreted by hypoxic lung cancer cells enhanced angiogenic potential and endothelial permeability^[66]^. We have shown that miR-1246 not only suppresses the expression of VE-cadherin, an adhesion molecule between ECs, but also induces the expression of ICAM-1, an adhesion molecule between tumor cells and ECs^[67]^. As research elucidates the mechanisms by which tumor-derived EVs affect ECs, it becomes clear that tumors establish a pre-metastatic niche via EVs.

## ACQUISITION OF DRUG RESISTANCE BY TUMOR EVS

### Induction of drug efflux pump expression by tumor EVs

**Figure 2:**
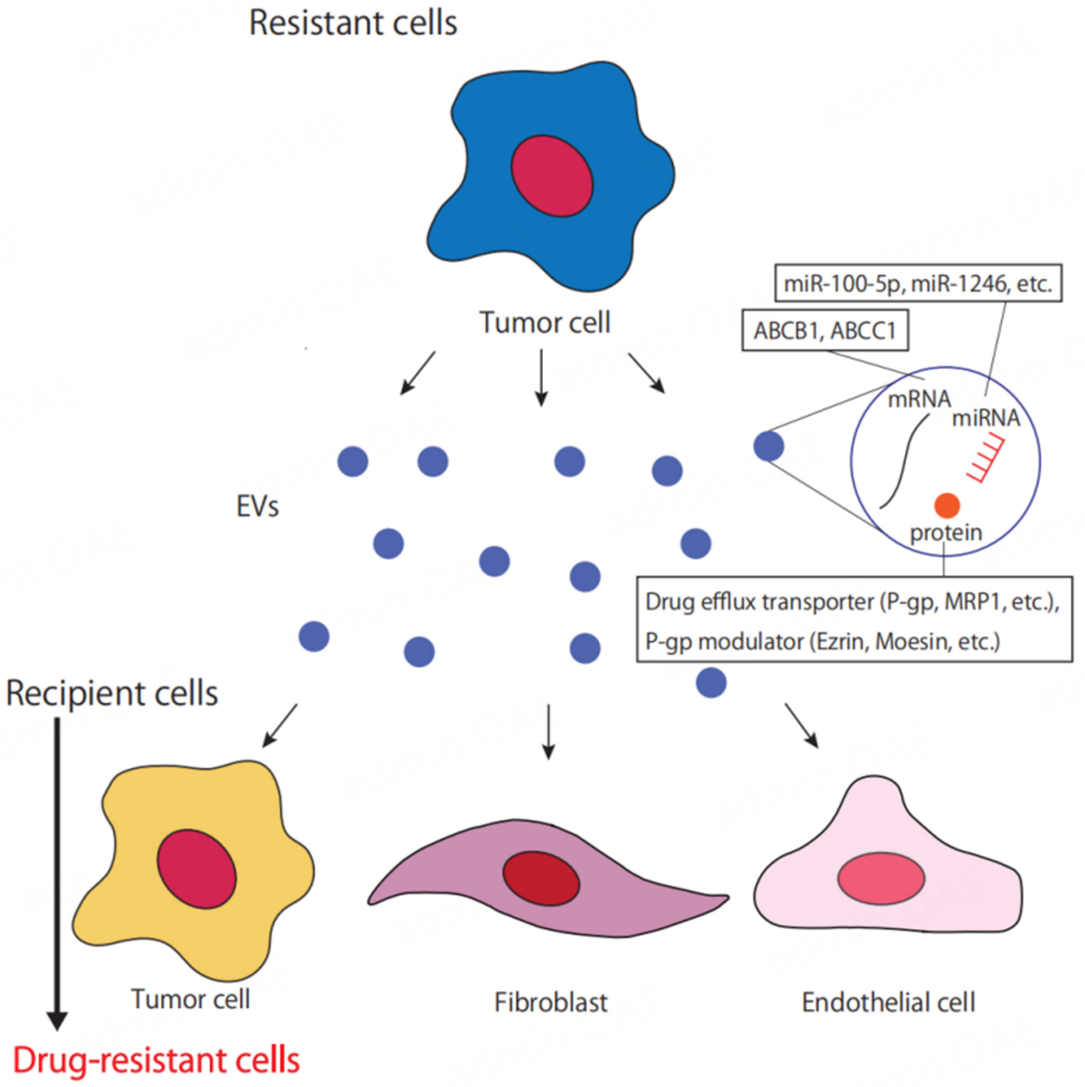
Drug resistance is conferred to other cells through tumor-derived EVs. Drug-resistant tumor cells confer drug resistance to recipient cells by transporting mRNAs for ABCB1 and ABCC1, miRNAs involved in signaling such as Akt, and drug efflux transporters and P-gp modulators via EVs. EVs: Extracellular vesicles; ABCB1: ATP-binding cassette subfamily B member 1; ABCC1: ATP-binding cassette subfamily C member 1; P-gp: P-glycoprotein.

Tumor-derived EVs are also closely associated with drug resistance^[68]^ [Figure 2]. There have been several cases in which tumor cells resistant to anticancer drugs confer drug resistance to sensitive cells via EVs. Qin *et al.* have reported that EVs derived from cisplatin-resistant lung cancer cells confer resistance to other sensitive cells^[69]^. In this study, they analyzed miRNA expression profiles of EVs derived from cisplatin-resistant cells and those from sensitive cancer cells, respectively. They focused on miR-100-5p, which is most downregulated in EVs derived from resistant lung cancer cells, and identified the mammalian target of rapamycin (mTOR) as a target gene using several bioinformatics methods. Using miR-100-5p mimics and inhibitors, they found that EVs derived from cisplatin-resistant cancer cells confer drug resistance via increased mTOR expression both *in vitro* and *in vivo*.

In addition, major proteins involved in the acquisition of multidrug resistance in tumor cells include P-gp/ABCB1, multidrug resistance-related protein1 [MRP1/ATP-binding cassette subfamily C member 1 (ABCC1)], and breast cancer resistance protein (BCRP/ABCG2)^[70]^. Increased expression of these transporters responsible for drug efflux in tumor cells promotes drug excretion, resulting in drug resistance. It has been reported that EVs secreted by drug-resistant tumor cells induce the expression of drug efflux pumps in drug-sensitive tumor cells, leading to drug resistance. EVs released from docetaxel-resistant tumor cells transport P-gp to sensitive cells and confer drug resistance^[71,72]^.

### Acquisition of drug resistance via EV-mediated packaging

Drug-resistant tumor cells are known to shed more EVs than drug-sensitive cells^[73]^. It has also been shown that exposure of tumor cells to therapeutic drugs promotes drug resistance by packaging drugs into EVs and releasing them outside the cell^[74]^. Rab27B is highly expressed in 5-FU-resistant hepatocellular carcinoma cells, which secrete large amounts of EVs upon 5-FU stimulation. Rab27B knockdown decreased EV secretion and increased intracellular accumulation of 5-FU^[75]^. Drug-resistant oral squamous cell carcinoma cells produce more EVs than drug-sensitive cells, and high levels of cisplatin are detected in the EVs. Inhibition of EV secretion by proton pump inhibitors resulted in increased drug sensitivity in cisplatin-resistant cells^[76]^. Exposure of multiple myeloma cells to the therapeutic drug lenalidomide has been shown to increase SORT1/LAMP2 expression and EV secretion, which in turn causes sensitive cells to acquire tolerance^[77]^. As shown above, drug packaging by EVs is one of the mechanisms for drug resistance, and research looking for its therapeutic application is currently ongoing.

### Acquisition of drug resistance in ECs by tumor EVs

**Figure 3:**
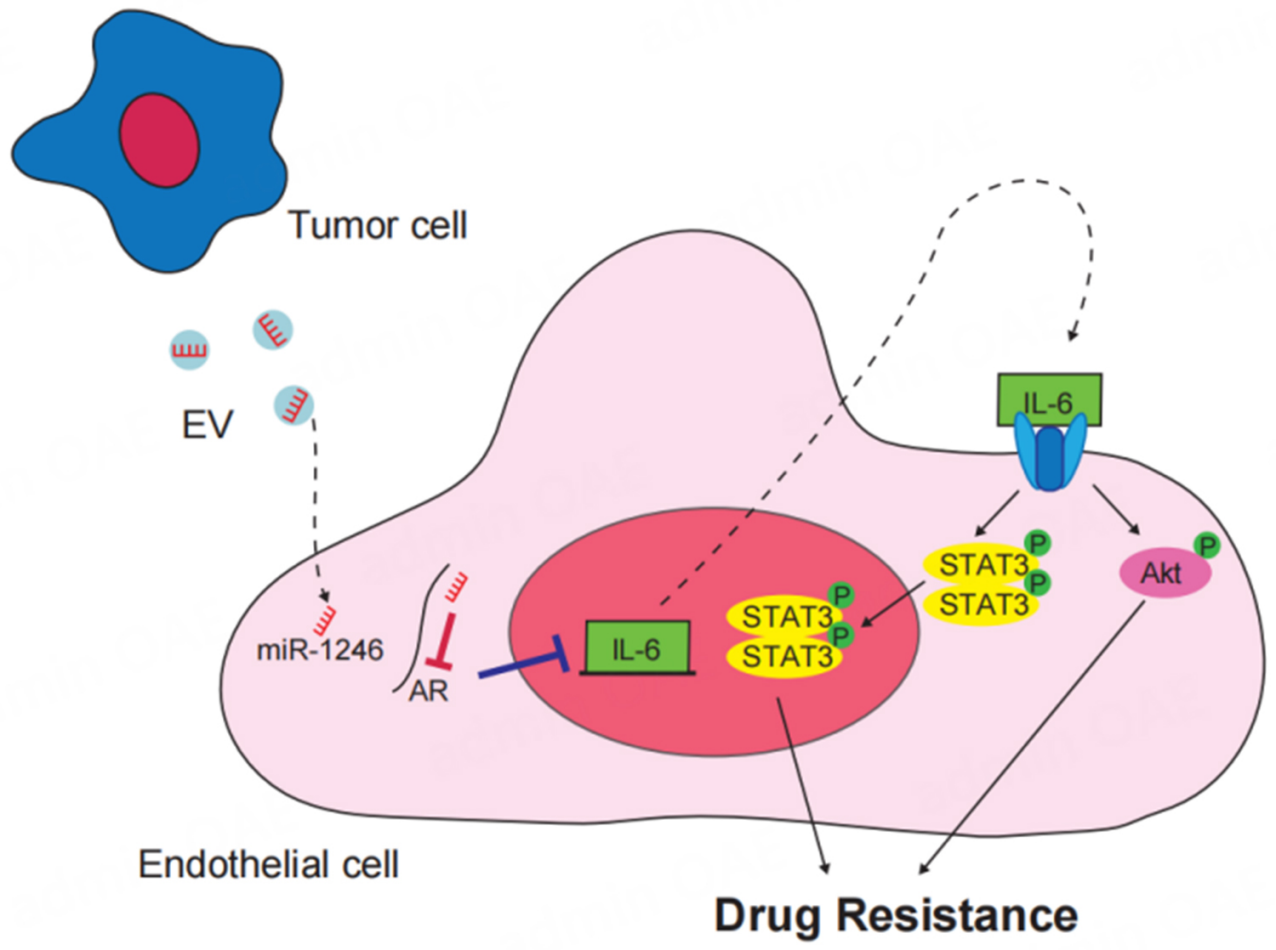
Drug resistance of ECs by tumor-derived EVs. Metastatic melanoma-derived EVs are taken up by ECs. Then, miR-1246 enhances IL-6 expression by targeting AR. An autocrine system is utilized to induce drug resistance via phosphorylation of Akt and activation of STAT3. This figure is a modification of the schema in^[78]^. ECs: Endothelial cells; EVs: extracellular vesicles; AR: androgen receptor; STAT3: signal transducer and activator of transcription 3.

Although it was thought that resistance to antiangiogenic therapy is primarily due to phenotypic changes of the tumor cells, as mentioned above, ECs also acquire drug resistance. Crosstalk between tumor cells and stromal cells is an important factor in tumor progression, and evidence is accumulating to support the idea that tumor cell-derived EVs are associated with drug resistance of ECs. Drug resistance of the TECs that make up tumor blood vessels means that pathways for nutrient and oxygen delivery to tumor cells and for metastasis remain. We have shown that VEGF-A induces MDR1/ABCB1 expression in NECs through activation of Y-box binding protein 1^[32]^. TECs were resistant to 5-FU, which is not a substrate of ABCB1^[39]^, suggesting that other mechanisms of drug resistance exist in TECs. We found elevations of IL-6 expression and Akt activation in TECs compared to NECs^[36]^, suggesting that tumor-derived EVs may act on this pathway as a tumor microenvironment factor. We analyzed miRNAs in EVs from high and low metastatic melanoma and identified that miR-1246 is abundant in EVs from highly metastatic melanoma^[78]^. Since IL-6 was not detected in the tumor-derived conditioned medium^[78]^, we hypothesized that the IL-6 autocrine loop in ECs receiving EVs was responsible for resistance. We then revealed the mechanism by which highly metastatic melanoma-derived EVs are taken up by ECs. In addition, miR-1246 targets the androgen receptor to increase IL-6 expression, leading to drug resistance via phosphorylation of Akt and signal transducer and activator of transcription 3 (STAT3)^[78]^ [Figure 3]. Dong *et al.* reported that TrpC5 and P-gp in breast cancer-derived EVs are transported to ECs, suggesting a mechanism of EC resistance^[79]^. However, drug resistance of ECs induced by tumor-derived EVs has not been reported elsewhere. Table 1 shows the reports of EC drug resistance by tumor EVs identified to date. Since EVs are complexes containing proteins and nucleic acids, it is expected that ECs may exhibit drug resistance by receiving drug efflux pumps and miRNAs that regulate molecular expression, but further analysis is needed.

**Table 1:** Reports on drug resistance of ECs by tumor-derived EVs

**Donor cell**	**Recipient cell**	**Relevant EV cargo**	**Target gene**	**Mechanism**	**Ref.**
A375SM cells	HMVECs	miR-1246	Androgen receptor	miR-1246 increases IL-6 expression by targeting the androgen receptor and activating Akt and STAT3	[78]
Adriamycin-resistant MCF-7 cells	HMECs	TrpC5, P-gp	-	TrpC5 incorporated into EC induces P-gp expression via translocation of NFATc3	[79]

ECs: Endothelial cells; EVs: extracellular vesicles; A375SM: highly metastatic melanoma cell; HMVEC: human dermis microvascular endothelial cell; STAT3: signal transducer and activator of transcription 3; MCF7: breast cancer cell; HMEC: human microvessel endothelial cell; P-gp: P-glycoprotein; TrpC5: transient receptor potential channel 5; NFATc3: nuclear factor of activated T cells isoform c3.

### Effects of TEC-derived EVs

Conversely, the effects of EVs derived from tumor stromal cells on tumor cells have recently been reported. EVs derived from cancer-associated fibroblasts (CAFs) have been shown to induce Snail expression, pancreatic cancer cell proliferation, and drug resistance^[80]^. Tumor-associated macrophage (TAM)-derived EVs were taken up by lung adenocarcinoma cells, and miR-942 was shown to promote the ability of invasion and migration by suppressing FOXO1 expression^[81]^.

There are also a few reports describing the effects of EC-derived EVs on tumor cells. miR-203 in human umbilical vein endothelial cell (HUVEC)-derived EVs inhibits the progression of non-small cell lung cancer (NSCLC) by targeting DTL and increasing the stability of p21, a tumor suppressor^[82]^. Lombardo *et al.* showed that IL-3 promotes EV release in ECs isolated from human umbilical veins and that miR-126-3p and pSTAT5 within these EVs induce angiogenesis in other ECs^[83]^. They found that a treatment with anti-IL-3R-α blocking antibody altered levels of miR-214-3p and miR-24-3p in TEC-derived EVs, and that TECs incorporating these EVs were involved in angiogenesis via the Wnt/β-catenin pathway^[84]^. Furthermore, IL-3R-α-inhibited and TEC-derived EVs reduced tumor cell viability and migration^[85]^.

Recently, TEC-derived EVs have been reported to inhibit tumor immunity. TEC-derived EVs from head and neck cancer acted on peripheral blood mononuclear cells (PBMCs) to modulate cytokine secretion and stimulate T regulatory cell (Treg) formation, thereby causing immunosuppression^[86]^. Some TEC-specific markers, such as biglycan, activated NF-κB, a transcription factor for the immune checkpoint molecule PD-L1^[26]^. Since EVs reflect the characteristics of the cells from which they are derived, TEC-derived EVs may have high PD-L1 expression and induce tumor immune evasion by inducing PD-L1 expression in tumor cells in a paracrine manner.

Currently, there are few reports on the effects of TEC-derived EVs, especially on tumor cells, and further research is needed.

## PROSPECTS FOR DRUG THERAPY FROM EV RESEARCH

Understanding the mechanisms of drug resistance of ECs via tumor-derived EVs or ECs themselves will lead to the development of new therapeutic strategies targeting both tumors and ECs to improve the outcome of patients with resistant tumors. Nishida-Aoki *et al.* have shown that the removal of tumor-derived EVs by intravenous administration of antibodies to tumor-bearing mice suppresses cancer metastasis^[87]^, suggesting a possibility of its therapeutic application. However, a method to target EVs for treatment has not yet been established for clinical uses. Discovery of a marker specific for EVs secreted by tumors and TECs will lead to an effective therapy.

In addition, recent evidence suggests that autophagy may contribute to the acquisition of drug resistance in ECs. Regarding bortezomib resistance in multiple myeloma, the combination of bortezomib, a proteasome inhibitor, and hydroxychloroquine, an autophagy inhibitor, was shown to promote autophagy of ECs and inhibit myeloma plasma cell growth^[88]^. These results demonstrate the efficacy of bortezomib in combination with an autophagy inhibitor in the treatment of resistant multiple myeloma, and indicate the necessity of using an angiogenesis inhibitor to achieve a complete remission. Therefore, we speculate that future research should focus on autophagy to further understand the mechanism underlying drug resistance of ECs.

## CONCLUSION

In the tumor microenvironment, EVs play an important role as signaling molecules between tumor cells and stromal cells. Tumor cell-derived EVs induce TEC-like characteristics in NECs, such as angiogenic potential and drug resistance. Overcoming drug resistance in TECs is crucial to improve therapeutic efficacy, since drug-resistant TECs promote tumor metastasis and support tumor cell proliferation. It is desirable to develop new therapies that target tumors and specifically TEC-derived EVs, while also elucidating the mechanisms underlying the acquisition of resistance.

## DECLARATIONS

### Copyright

© The Author(s) 2024.
